# Case Report: Initial Evidence of Safety and Efficacy of High Definition-Transcranial Direct Current Stimulation in a Patient With Neuropathic Pain and Implanted Spinal Cord Stimulator

**DOI:** 10.3389/fpain.2021.753464

**Published:** 2021-11-18

**Authors:** Choi Deblieck, Steven Smeijers, Bart Morlion, Abhishek Datta, Chris Thomas, Tom Theys

**Affiliations:** ^1^Academic Center for Electroconvulsive Therapy (ECT) and Neuromodulation, University Psychiatric Center, KU Leuven, Leuven, Belgium; ^2^Department of Neurosurgery, University Hospitals Leuven, Leuven, Belgium; ^3^Department of Cardiovascular Sciences, Section Anaesthesiology & Algology, KU Leuven-University of Leuven, Leuven, Belgium; ^4^Research and Development, Soterix Medical Inc., Woodbridge, NJ, United States; ^5^Department of Neurosurgery, UZ Leuven, Leuven, Belgium; ^6^Biomedical Engineering, City College of New York, New York, NY, United States

**Keywords:** neuropathic pain, high definition transcranial direct current stimulation, modeling, complex regional pain syndrome, non-invasive electrical stimulation

## Abstract

Neuropathic pain (NP), often treatment-refractory, is one of the most debilitating conditions contributing to suffering and disability worldwide. Recently, non-invasive neuromodulation techniques, particularly repetitive transcranial magnetic stimulation (rTMS) and transcranial direct current stimulation (tDCS) have emerged as potential therapeutic alternatives due to their ability to alter cortical excitability of neural circuits. However, the magnetic field induced in rTMS may be unsafe for patients with an implanted electrode in the head or neck area while tDCS poses no theoretical risk of injury to these patients. High definition (HD)-tDCS is a novel, more focal technique of tDCS and may be safer to the patient compared to the more diffuse stimulation of conventional tDCS. To our knowledge, no study has ever demonstrated the safety and/or feasibility of HD-tDCS in patients with spinal cord stimulation (SCS) devices using computational modeling of induced electrical fields. Furthermore, this study highlights the potential use of (HD-)tDCS as predictive tool for a positive response in chronic epidural motor cortex stimulation (MCS), especially in patients with an implanted device not suitable for rTMS. In a 54-year-old woman with an implanted spinal cord stimulation (SCS) system for another pain syndrome, HD-tDCS was initiated for refractory post-surgical inferior alveolar nerve neuropathy. She was submitted to 7 days of anodal HD-tDCS over the left motor cortex at 1.5 mA for 30 min. A notable decrease in pain perception was observed, lasting for approximately 5–6 h (Numeric Rating Score decreased from 8 to 4.34). No adverse events were reported. The stimulation parameters and clinical efficacy of the SCS system remained unchanged. Additionally, computational analysis indicated no meaningful alteration of current flow when considering a model with a SCS implant with respect to a model without implant. Regarding the positive therapeutic effect of HD-tDCS, the patient was selected for an epidural MCS trial and subsequent implantation, which showed short-term pain relief of 50–75%. Although one case does not demonstrate efficacy, tolerability, or safety to the novel intervention, it paves the way for better diagnosis and treatment for patients who are otherwise excluded from other non-invasive neuromodulation techniques, such as rTMS. A positive tDCS effect could be a potential biomarker for positive epidural MCS response in patients with an implanted stimulation device non-compatible with rTMS.

## Introduction

Neuropathic pain (NP) is one of the most debilitating conditions contributing to suffering and disability worldwide. Unfortunately, NP can be very treatment-refractory, with many patients continuing to report significant pain and repercussions on quality of life (QoL) despite the recommended multimodal pharmacological and non-pharmacological management, including physical therapy and cognitive behavioral therapy. In recent years, non-invasive brain stimulation techniques have emerged as potential therapeutic alternatives, next to invasive neuromodulation strategies such as epidural motor cortex stimulation (MCS) and deep brain stimulation (DBS). Indeed, two non-invasive methods have received increased interest, i.e., transcranial direct current stimulation (tDCS) and repetitive transcranial magnetic stimulation (rTMS). The magnetic field induced in rTMS may be unsafe for patients with an implanted electrode in the head or neck area while tDCS poses no theoretical risk of injury to these patients. Cortical modulation by tDCS may increase glutamine and glutamate under the stimulating electrode, have effects on the μ-opioid receptor, and restore impaired intracortical inhibition (1). The analgesic benefits of tDCS can occur both during stimulation and beyond the time of active stimulation (1).

High definition (HD)-tDCS may be a safer therapeutic option compared to the more diffuse stimulation of conventional tDCS. HD-tDCS is a novel approach that uses arrays of smaller electrodes (2, 3) that are placed in a configuration that can be optimized for targeting (4, 5). In particular, the 4x1-ring montage of HD-tDCS has been proposed for unidirectional and targeted stimulation, with the polarity (anode or cathode) set by a center electrode and the area of cortical modulation restricted by adjusting the radii of 4 return electrodes (6). This configuration can not only achieve beneficial clinical effects with larger effect sizes more comparable to invasive interventions such as epidural MCS for chronic pain (7) but it may also enhance the understanding of the target cortical regions involved in interventions not easily attained by standard diffuse tDCS. Furthermore, increased focal intervention allows for tailoring of stimulation to individual indications and symptoms and may also reduce potential side effects due to decreased stimulation of neighboring regions, making it a safer alternative (8).

Few studies have been published to date assessing its effects in a patient population with respect to pain (8–12). Patients with preexisting implants, and especially head implants are, by default, excluded for tDCS as a precautionary measure (13). However, a large electrode impedance is expected between the metal implant and the surrounding tissue which makes it very unlikely for significant current flow across the metal implant (14) since the metal inside the body, with a high nominal conductivity, is an electron current carrier whereas current carried by tDCS through the body is ionic (13). Thus, there is no theoretical risk of injury to a patient with a pre-existing implanted neurostimulator based on modeling (13), as Houde et al. (15) were to first to demonstrate in their 37-year-old female patient with left lower limb complex regional pain syndrome (CRPS) and implanted spinal cord stimulation (SCS). Their results reported the efficacy of combined tDCS and transcutaneous electrical nerve stimulation (TENS) treatment, but not of tDCS as a stand-alone treatment.

Motor cortex stimulation (MCS) has been proposed as an invasive neuromodulation technique for chronic refractory NP since the early 1990s and with variable treatment results, for patients for whom no other treatment modality is available. In MCS, an epidurally placed electrode delivers a targeted current to the very closely located precentral primary motor cortex (M1). MCS literature suffers from low level of evidence due to small retrospective series, substantial inter-study heterogeneity and confounders, making correct interpretations difficult. A systematic review concluded it to be relatively safe and effective with a “good outcome” (≥40–50% pain improvement) in 56.7% of patients (16). A recent double-blind, sham-controlled randomized trial of 18 subjects found a clinically relevant response in approximately 40% of patients and 39% were long-term responder (17). The probability of reducing NRS scores by ≥2 points or by any amount compared to sham stimulation was 41.4 and 88.6%, respectively. Strikingly, as seen in deep brain stimulation for movement disorders, 39% of patients had an “insertional effect” consisting of a substantial postoperative analgesic effect in the absence of stimulation for at least a few weeks, and those with an insertional effect showed almost 100% probability of having a response to MCS. In the light of these results, preoperative prediction of a beneficial response is increasingly important since only a subpopulation of chronic neuropathic pain patients responds to MCS. Some identified predictive factors are type of pain syndrome (facial pain, phantom limb pain, and CRPS, as compared to poststroke pain and brachial plexus lesions), positive insertional effect (17) and response to rTMS (16, 18, 19). MCS significantly improved quality of life (QoL), notably without correlation between NRS reduction and QoL measures, but these measures remain underexposed (20).

To the best of our knowledge, HD-tDCS with superior focality, more comparable to epidural MCS, has never been applied in patients with cortical or cervical SCS implants. In order to evaluate the analgesic effects of HD-tDCS in a patient with neuropathic pain with a cervical epidural electrode, we submitted our patient to 7 days of active anodal HD-tDCS to determine if HD-tDCS (1) is a safe and effective therapeutic alternative for patients with a cervical electrode implant, capable of decreasing pain perception, and (2) may serve as a valid and reliable method to select eligible patients for epidural MCS who are otherwise excluded from other non-invasive brain stimulation techniques as a pre-operative tool.

## Case Presentation

A 54-year-old woman presented with uncontrollable neuropathic pain in the lower right side of her face. Three years prior, she received an SCS system to treat CRPS, after sustaining a post-operative axillary nerve lesion. The tip of the electrode was positioned a C2 which resulted in a good pain reduction. Two months prior to HD-tDCS, she underwent apex resections of teeth 25 and 47, complicated by pain and wound infections. A computed tomography (CT) scan showed the remainder of the root of tooth 47 was in contact with the mandibular canal. After revision surgery, the pain remained unabated with a Visual Analog Score (VAS) score of 9.5. In addition, she experienced sleeping difficulties and weight loss. Her pain was triggered by chewing or changes in temperature. Clinical examination showed hypoesthesia and allodynia over the mandibular nerve region (V3), resulting from a post-surgical right inferior alveolar nerve injury. The DN4 (Douleur Neuropathique 4 Questions) questionnaire was 8, suggesting neuropathic pain diagnosis. Multiple trigger points over the right masseter muscle could elicit her typical pain pattern.

She was treated at another hospital with pulsed radiofrequency to the right mandibular nerve without effect. Her medication included Paracetamol, Methadone, Clonazepam & Ketorolac. She was referred to our tertiary care center, University Hospitals Leuven, for further treatment.

## Intervention

Our patient, RT, received 7 daily sessions on consecutive business days (from March 17 to March 24, 2017). We employed a 4x1 Multichannel Stimulation Adaptor (Model 4x1-C2, Soterix Medical Inc., New York, NY) connected to a conventional 1 × 1 tDCS device (Model 1224- B, Soterix Medical Inc.) to deliver direct current to the scalp via HD electrodes. Electrodes were held in place by specially designed plastic casings embedded in a modular electroencephalography (EEG) recording cap. We positioned the center anodal electrode over C3 based on the International 10/20 EEG System (48) which corresponds approximately to the location of the left M1. Four return cathodal electrodes were placed in a radius of approximately 7 cm from the center electrode. Their locations corresponded roughly to Cz, F3, T7, and P3. The hair underlying each electrode was separated as to expose the scalp, and approximately 1.5 mL of highly conductive gel (Signa Gel, Parker Laboratories, Fairfield, NJ) was placed beneath each electrode to improve conductance. Given that electrode resistance is nonlinear to electrode-interface electrochemical processes (14) electrode resistance (impedance) can be misleading. For example, the resistance apparently measured fully depends on test current (21). Therefore, based on prior experience in set-up and stimulation using HD electrodes, contact quality is normalized to “quality units” by the 4x1 Adapter test circuit. Impedance values were verified to be 1.50 “quality units” for each of the 5 electrodes before the beginning of each stimulation session. During each active 4x1-ring HD-tDCS session, DC was gradually ramped up over a period of 30 s until reaching an intensity of 1.5 milliamperes (mA), which were delivered for 20 min on the first 3 days and increased to 30 min on days 4–7 of the treatment. These parameters previously showed to be well-tolerated in healthy subjects (1, 22). The stimulation parameters of her implantable pulse generator (IPG) remained unchanged on the days of the intervention and post treatment (stimulation amplitude, pulse frequency, pulse width, and used anodal/cathodal contacts in different stimulation programs). Before each HD-tDCS session, RT turned off her SCS IPG and turned it back on after the session. Furthermore, the therapeutic effect of SCS on her CRPS complaints remained unchanged after HD-tDCS.

**Figure 1:**
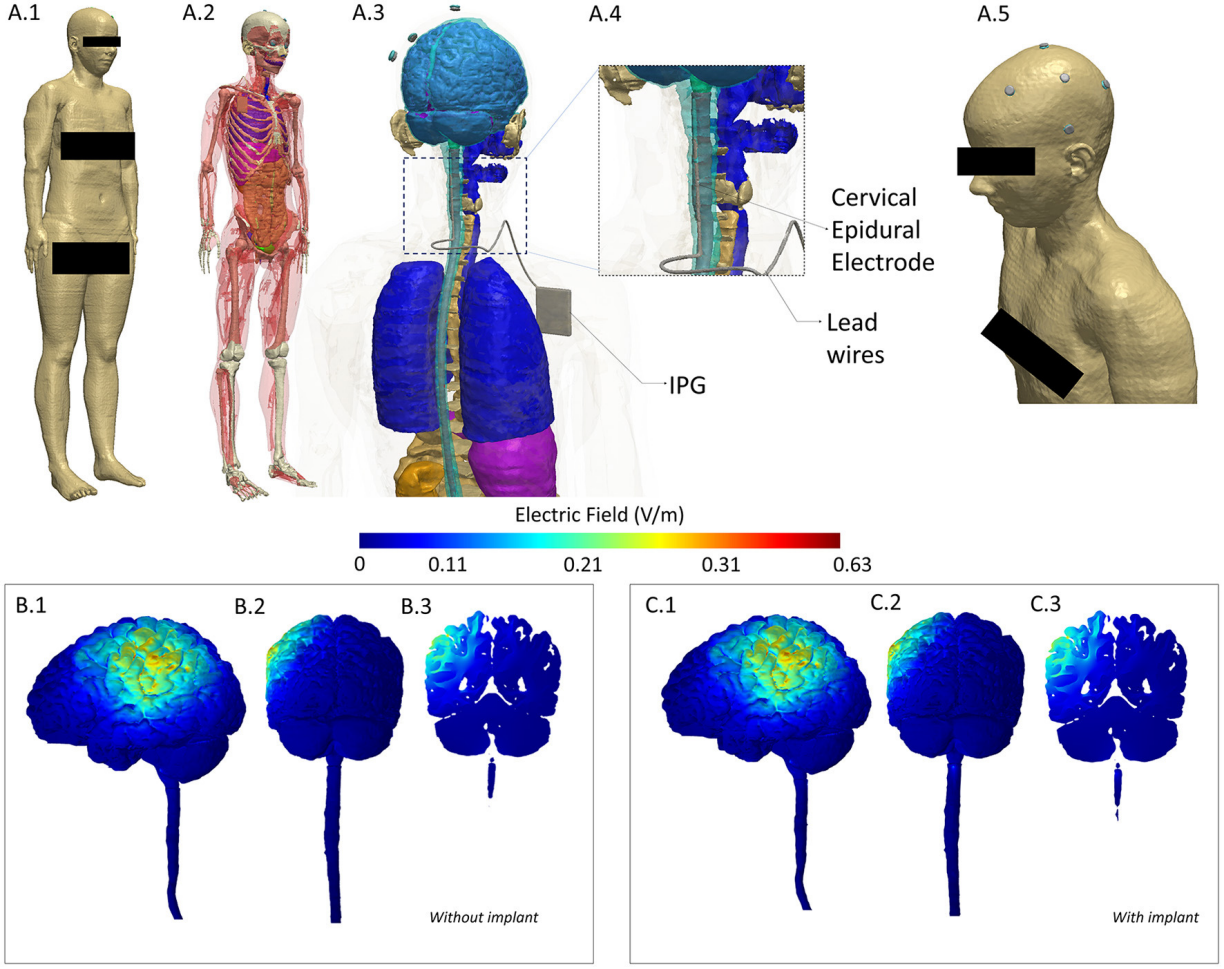
Computational Model of induced electric field to evaluate any alteration due to implant. To investigate deviation (intensity or location of current flow), we considered a model derived from a healthy subject (no implant) and incorporated a spinal cord implant within the same geometry to serve as our implant case. We performed a comparison of induced electric field between the two models and noted a difference of 1.1% in maximum and no difference in mean and median values. **(A.1)** A full body head model was derived from the Visible Human Project (24). A female dataset was used given the gender of the patient. **(A.2)** Skin mask is made transparent to reveal tissue segmentation detail. **(A.3)** Close-up of the model along with geometry of the Spinal Cord Stimulation (SCS) considered. **(A.4)** The cervical section of the model is expanded to highlight the location of the epidural electrodes and lead wires. **(A.5)** Skin mask along with stimulation electrodes indicating the montage simulated replicated anodal HD-tDCS over left M1. **(B.1–B.3)** 3D Left Lateral, 3D Posterior, and 2D coronal plots of induced electric field in the brain and in the spinal cord for the healthy (no implant) situation. **(C.1–C3)** Corresponding views for the implant situation. Results indicate no alteration in current flow—at left M1 (both at cortical surface and in deeper areas) and in overall pattern. Further, posterior plots indicate no deviation locally at the implant level (see the spinal cord section in **B.2,C.2**). All methods and analyses were based on prior work by our group (25–27). A current injection of 1.5 mA and the classic Laplace's equation was used to determine induced current flow.

Outcome measures were: (1) Numeric Rating Scale (NRS) assessed daily, (2) the Short Form-36 Dutch version (SF-36), and (3) the MPQ-DLV Pain Questionnaire, the Dutch version of the McGill Pain Questionnaire (MPQ) (23), both assessed weekly. We also performed a finite element based computational analysis to investigate any deviation in current flow pattern due to incorporation of a SCS implant in a realistic human model (Figure 1).

The *NRS* is a single 11-point numeric score in which a patient rates their pain by selecting a whole number (0–10 integers) that best represents the intensity of their pain (28) with 0 representing one pain extreme (e.g., “no pain”) and 10 representing the other pain extreme (e.g., “pain as bad as you can imagine” and “worst pain imaginable”) (28, 29). The NRS provide an estimate of patients' pain intensity and can be assessed verbally or in writing. RT was asked to rate her pain at six and 10 in the morning, at 2 and 6 in the afternoon, at 10 in the evening, and at night during the treatment. Daily baseline scores were recorded over a week. Both the baseline and post HD-tDCS Daily NRS scores were averaged to obtain a baseline and post treatment score.

Both the SF-36 and MPQ-DLV were assessed weekly. The former is a health status profile comprising 36 questions that reflect 8 domains of health, including physical functioning, physical role, pain, general health, vitality, social function, emotional role, and mental health (30), and has been found to be a reliable and valid questionnaire for measuring health-related quality of life of individuals with several chronic health conditions (31). Lastly, the MPQ-DLV is a multidimensional pain questionnaire comprising 2–6 words that fall into 4 subscales evaluating the sensory (-S), affective (-A), and evaluative (-E), and miscellaneous aspects of pain (29, 32). In addition, there is also a 1-item pain intensity scale (32). The MPQ is a generic pain measure useful to evaluate both the quantity (intensity) and the quality of how patients assess their pain (29).

On October 10, 2017, after performing non-invasive transcranial stimulation using tDCS, the patient was selected for an invasive epidural MCS trial for 4 weeks. During a neurosurgical procedure with neuronavigation, a plate-electrode (Medtronic Specify 5-6-5, Minneapolis, Minnesota) was epidurally advanced over the precentral primary motor cortex (M1) and electrode leads were externalized. Electrode position was verified using intra-operative neuromonitoring techniques of somatosensory evoked potentials (SSEPs) with phase reversal and motor evoked potentials (MEPs) with cortical stimulation, the latter which halted contractions in the painful facial area. During the trial, pain scores (NRS) and quality of life measures were rated daily. After definite MCS implantation, follow up was organized four-weekly in the first 3 months, whereafter follow-up was extended to every 2–3 months in the first year. During clinical visits, NRS was recorded, potential side effects were monitored, and stimulation parameters were reprogrammed when needed.

## Simulation to Evaluate Potential Alteration of Current Flow Due to Implant

To investigate potential deviation (intensity or location of current flow) due to the implant, we considered a whole-body female model (“Ella”) from the Virtual Family dataset (24). We developed a finite element method (FEM)-based computational model based on extensive prior work by our group (2). Briefly, tissue compartments were first modified to ensure continuity and compartments with same electrical conductivity merged to simplify the model (Simpleware-Synopsys Ltd., USA). The SCS implant was modeled as having three essential components (electrode, lead wires, and an implantable pulse generator) in a Computer Aided Design (CAD) software. The implant was then integrated within the segmented image data simulating the exact location (cervical) as present in the patient to serve as our implant case. In addition, we also considered a model with no implant to serve as the without implant case. Stimulation electrodes and conductive gel were also imported as CAD files and integrated within the scalp tissue compartment in both models (with and without implant) mimicking the exact electrode placement as used experimentally. Volumetric meshes were then generated from the data and exported to a FEM solver for computation (COMSOL Multiphysics, USA). All compartments (tissues, SCS implant, electrodes, and gel) were assigned representative electrical conductivity values and boundary conditions imposed reflecting transcranial stimulation using direct current. The standard Laplacian equation was solved and the induced electric field (EF) magnitudes were determined (6). We plotted the EF in 3D and 2D views to facilitate a visual comparison between the two cases. We also quantified potential differences in terms of maximum, mean, and median EF.

## Results

After an initial 3-day-trial-and-error period, our patient, RT, showed a gradual and progressive improvement on the NRS (Table 1) and SF-36 (Table 2). In contrast, the MPQ-DLV only showed an overall improvement on the QoL (Quality of Life) subscale while the subscales NWC-T (Total Number of Words) or PRI-T (Totel Pain Rating Index) did not show any improvement (Table 3). A significant decrease in pain perception was perceived almost instantly, starting at approximately 5 min into the stimulation and lasting for approximately 3–6 h after the end of the treatment. Throughout the intervention period, RT could sleep through the night without having to depend on her nightly dose of Xanax (0.5 g). After three sessions, RT could touch her right jaw without feeling the usual pain. The patient did not experience any adverse events.

**Table 1:** NRS.

**Mean scores (7 days)**	**Baseline**	**Post HD-tDCS**
**Morning**		
6 a.m.	2	1.67
10 a.m.	5	5.5
**Afternoon**		
2 p.m.^*^	7	4.17
6 p.m.	9	4.5
**Evening**		
10 p.m.	7	4.6
**Night**	5	0

* *Patient received treatment at 1 p.m. Maximal effect for 5–6 h post treatment*.

**Table 2:** SF-36.

**Scores items to form**	**Baseline**	**Post HD-tDCS**
Physical functioning	50	100
Role functioning/physical	0	0
Role functioning/emotional	0	0
Energy/fatigue	0	200
Emotional well-being	64	280
Social functioning	0	75
Pain	0	40
General health	20	100

**Table 3:** MPQ-DLV.

	**Baseline**	**Post HD-tDCS**
**NWC-T**	19	19
NWC-S	9	8
NWC-A	6	5
NWC-E	4	6
**PRI-T**	40	42
PRI-S	15	15
PRI-A	12	9
PRI-E	13	18
**QoL**	25	19

Similar to the tDCS results, the 4-week MCS trial halted an average pain relief of 50–75% (NRS). Therefore, a second procedure with internalization of the leads and IPG placement was performed 1 week after the trial. The beneficial effect of MCS, however, lasted only 3 months. Despite extensive follow up with multiple reprogramming efforts, which only resulted once in intermittent, non-lasting pain relief at 11 months postoperatively (NRS 4) (stimulation amplitude of 60% of the motor threshold for facial contractions), no effect of MCS persisted after 1 year (NRS 8). There were no adverse effects of MCS. The computational model simulating transcranial application is an implant indicated a difference of 1.1% in the maximum EF with respect to the without implant case (Table 4). There was no difference noted in case of mean and median EF. Furthermore, we noted no difference in the induced cortical EF patterns in representative 2D and 3D views.

**Table 4:** e-field values.

	**Max EF (V/m)**	**Mean EF (V/m)**	**Median EF (V/m)**
With implant	0.624	0.024	0.051
Without implant	0.631	0.024	0.051

## Discussion

For RT received 7 daily sessions on consecutive business days. Evidently, we cannot eliminate a placebo effect or the possibility that our patient experienced a significant decrease in pain perception unrelated to HD-tDCS. However, it seems highly unlikely that uncontrollable facial neuropathic pain felt on the lower right side of her face, with a NRS score of 8 pre-intervention, decreased spontaneously and gradually to a 4.34 in the period of maximal effect (i.e., 5 h after stimulation or between 2 and 6 p.m.). Additionally, she notably improved on the SF-36 (except for the two Role Functioning subscales, physical and emotional). The overall QoL subscale of the MPQ-DLV (from a 25 to 19) also showed some improvement while the NWC-T and PRI-T showed no difference between pre and pre and post intervention. A site-specific effect in sensory and pain threshold modulation was observed following an increase in the excitability of M1/primay sensory (S1) in healthy individuals and M1/dorsolateral prefrontal cortex (DLPFC) in patients with chronic pain (33). Stimulating M1 may increase the activity of the insula and thalamus (34). Consequently, the insular-thalamic pathway activation following a-tDCS of M1 may have modulated RT's sensory/pain threshold (QoL) while leaving appraising words describing her state best unchanged (MPQ-DLV). Equally, a bigger magnitude of stimulus may have been required to induce a perception response (34, 35). In our case report, we carefully increased the current from 1.5 to 2 mA and the duration from 20 to 30 min in the course of the seven sessions. As a result, more sessions of HD-tDCS at 2 mA for 30 min may have been detrimental in order to generate a neuromodulatory effect high enough to affect the NWC-T and PRI-T subscales of MPQ-DLV. Furthermore, different forms of pain measures were used. For example, self-rating one's own perception of QoL requires different brain areas and networks relative to determining which word describes best to sensory and affective states. This may be in line with the observation that a-tDCS over M1 may have a superior therapeutic effect on the lower limbs relative to face and hand areas (10). Thus, the effect of tDCS in other brain areas, such as the DLPFC or S1, may provide different results (35, 36) since M1 had no significant relationship with the NWC-T and PRI-T subscales of MPQ-DLV. In addition, various factors (e.g., anatomical and functional network variations, psychological and neurophysiological states, receptor sensibility and neurotransmitter levels, etc.) could affect how one responds to tDCS (36).

Studies in the literature that employed non-invasive neuromodulation in chronic pain patients with a permanent implanted electrode with the more focal and novel method of HD-tDCS are non-existent. Only one recent case report demonstrated that applying conventional anodal tDCS at 2 mA for 25 min over right primary motor cortex (C4) in a patient with left lower limb CRPS and implanted SCS device was feasible and safe (15). However, only when tDCS was combined with TENS, pain intensity and unpleasantness were slightly reduced. A review paper on conventional tDCS reported that clinical trials in patients with NP apply 1–2 mA anodal tDCS over the primary motor or DLPFC for 20 min for 5 consecutive days (1). These trials observed a significant decrease in pain perception that lasted between 2 and 4 weeks after treatment. No severe adverse events were reported. In our patient, for the sake of safety, the current of 1.5 mA on days 1 to 3 was increased to 2 mA on days 4 to 7 after no adverse events were reported. The duration of stimulation was prolonged on day 4 from 20 to 30 min. The configuration of the more novel and focal method of HD-tDCS with greater magnitude of its aftereffects (36) was the preferred treatment in our patient due to two of its advantages: its beneficial clinical effects with larger effect sizes is not only more comparable to invasive interventions such as epidural M1 stimulation for chronic pain (7) but it may also reduce potential side effects due to decreased stimulation of neighboring regions, making it a safer method (8).

Our patient's steady and progressive improvement over the course of treatment implicates that with each additional treatment session, the gradual neuromodulatory effects generated by tDCS may have resulted in an additive, incremental functional brain reorganization in the motor cortex (37). The neurobiological effect of tDCS in patients with neuropathic pain points toward dysfunctional intracortical inhibition (1). Since tDCS produces a weak, stable, electric current, it has been suggested that anodal tDCS may alleviate neuropathic pain symptoms through a change in membrane resting potential, particularly a depolarization of the stimulated area (38), and thus normalizing impaired neural activity. The effects that last beyond stimulation may involve other mechanisms such as the synaptic transmission modulation through NMDA receptors (39). The increased cortical excitability after anodal HD-tDCS resulting in a decrease in pain, may be associated with an up-regulation of neural activity in the motor cortex. As a result, the perception of pain may be altered indirectly though neural circuits of pain-modulating areas, specifically the thalamic nuclei (1). A neuroimaging study that explored the activity of the motor cortex stimulated with epidural electrodes demonstrated changes in activity in thalamic and subthalamic nuclei (40). It has been argued that activation in the thalamic nuclei may affect other pain-related brain areas (e.g., anterior cingulate, peri-aqueductal gray, spinal cord) with the outcome of a change in the affective-emotional component of pain and inhibition of pain impulses from the spinal cord (41).

In contrast to the case report by Houde et al. (15), our patient reported a beneficial effect of HD-tDCS that gradually increased slightly with each additional session from 5 min into the stimulation to 6 h after the seventh and last treatment session. Although conventional tDCS has been reported to be more effective in the treatment of lower limb neuropathic pain (10), HD-tDCS, being more focal and less diffuse, may have been more effective in our patient with facial neuropathic pain on the lower right side of her face, and this more similar to the more focal stimulation of rTMS with “level A of definite analgesic effect.” Similarly, less favorable efficacy of primary motor rTMS has been observed in lower limb neuropathic pain relative to face or upper limb neuropathic pain for (42, 43). It should be noted that Houde et al. (15) did not include a TENS only condition and that the time interval between tDCS and TENS may have been too short to differentiate the analgesic effect of tDCS alone from the potential synergetic effect of both interventions. Furthermore, tDCS was not used as a biomarker in the setting of patient selection for chronic epidural MCS.

Our study did not evaluate the therapeutic effects of HD-tDCS on M1 modulation beyond 6 h. Further studies are required to improve our understanding of the acute effects of HD-tDCS and the effects over time by systematically studying number of sessions, time, current intensity, and electrode size. Further, systematically searching for predictors/biomarkers (e.g., neuropsychological, neurophysiological) and optimal state/conditions (e.g., psychotherapy, pharmacotherapy) associated with tDCS remains crucial. Furthermore, future studies should compare the effect of tDCS over different areas of stimulation (e.g., DLPFC vs. M1) while selecting different pain outcome measures as to better understand the responses following conventional tDCS or HD-tDCS. Finally, since pain studies predominantly use subjective outcome measures, it is paramount to include a sham condition in clinical studies and settings to warrant complete blinding.

This case study highlights the potential use of non-invasive HD-tDCS as preoperative predictive tool for invasive MCS therapy, especially in patients with an implanted device. Indeed, the beneficial effect of HD-tDCS was reproduced in the epidural MCS trial with 50–75% pain relief. However, the patient showed no long-term response despite multiple reprogramming efforts (only an intermittent, non-lasting effect 10 months postoperatively was seen), for reasons that remain elusive. The short-term MCS effect in this case cannot be seen as the “insertional effect” as reported in Hamani et al., since stimulation was immediately started postoperatively. Another non-invasive strategy, rTMS, has already been associated with positive MCS effect in some series and could serve as a potential biomarker (18, 19). Future studies are needed to confirm the use of (HD-)tDCS for this purpose.

Our case report demonstrates the safety and short-term efficacy of HD-tDCS treatment in a patient with an implanted SCS system. TMS, usually the preferred method of non-invasive neuromodulation (10, 12, 43, 44, 47), may be considered unsafe in these patients due the induced magnetic field (12, 45, 46). Additionally, computational analysis of induced electric field indicated no difference in mean and median values and a 1.1% difference in maximum value when comparing a model without implant to a model with a SCS implant. Given the placebo effects of pain treatments in patients with neuropathic pain in general, the results of our single patient, RT, should be interpreted with caution. Nonetheless, our results of anodal HD-tDCS on RT on the safety of the procedure and acute beneficial effect is encouraging and invites future studies to consider HD-tDCS as an alternative treatment option of neuropathic pain patients with or without head/neck implants in both the research and clinical settings, and as a preoperative screening tool for epidural MCS in this patient population.

In conclusion, our findings, together with the similar case report using conventional tDCS in a patient with an implanted SCS device, confirms the need for sham-controlled clinical trials of (HD)-tDCS as a potential pre-operative therapeutic tool in patients with permanent electrode implants in the head area who are candidates for surgically implanted chronic epidural MCS. In addition, determining the ideal dose, duration, sessions, and maintenance protocol of tDCS remains crucial in optimizing tDCS efficacy to reduce pain intensity and maximize the QoL of patients with chronic neuropathic pain. Future studies are needed to determine if HD-tDCS could serve as an add-on treatment or biomarker to select eligible patients for MCS.

## Data Availability Statement

The raw data supporting the conclusions of this article will be made available by the authors, without undue reservation.

## Ethics Statement

Ethical review and approval was not required for the study on human participants in accordance with the local legislation and institutional requirements. The patients/participants provided their written informed consent to participate in this study.

## Author Contributions

CD wrote the manuscript, treated RT using HD-tDCS, and evaluated the acute effect of HD-tDCS. SS was responsible for the MCS data and manuscript writing and revision. BM and TT interpreted the data and revised the manuscript for intellectual content. AD and CT performed the modeling and simulation analysis. All authors participated in editing.

## Conflict of Interest

AD and CT were employed by Soterix Medical Inc. The remaining authors declare that the research was conducted in the absence of any commercial or financial relationships that could be construed as a potential conflict of interest.

## Publisher's Note

All claims expressed in this article are solely those of the authors and do not necessarily represent those of their affiliated organizations, or those of the publisher, the editors and the reviewers. Any product that may be evaluated in this article, or claim that may be made by its manufacturer, is not guaranteed or endorsed by the publisher.
